# A Perspective on Secondary Seed Dormancy in *Arabidopsis thaliana*

**DOI:** 10.3390/plants9060749

**Published:** 2020-06-15

**Authors:** Gonda Buijs

**Affiliations:** Department of Botany and Plant Biology, University of Geneva, CH-1211 Geneva, Switzerland; gonda.buijs@unige.ch

**Keywords:** secondary seed dormancy, dormancy cycling, germination arrest, abscisic acid, seed dormancy, environmental factors, *Arabidopsis thaliana*

## Abstract

Primary seed dormancy is the phenomenon whereby seeds newly shed by the mother plant are unable to germinate under otherwise favorable conditions for germination. Primary dormancy is released during dry seed storage (after-ripening), and the seeds acquire the capacity to germinate upon imbibition under favorable conditions, i.e., they become non-dormant. Primary dormancy can also be released from the seed by various treatments, for example, by cold imbibition (stratification). Non-dormant seeds can temporarily block their germination if exposed to unfavorable conditions upon seed imbibition until favorable conditions are available. Nevertheless, prolonged unfavorable conditions will re-induce dormancy, i.e., germination will be blocked upon exposure to favorable conditions. This phenomenon is referred to as secondary dormancy. Relative to primary dormancy, the mechanisms underlying secondary dormancy remain understudied in *Arabidopsis thaliana* and largely unknown. This is partly due to the experimental difficulty in observing secondary dormancy in the laboratory and the absence of established experimental protocols. Here, an overview is provided of the current knowledge on secondary dormancy focusing on *A. thaliana*, and a working model describing secondary dormancy is proposed, focusing on the interaction of primary and secondary dormancy.

## 1. Introduction

Seeds comprise an important phase in the life cycle of seed plants, as they are the next generation of the plant and can be widely dispersed. Subsequent seed germination and seedling establishment are crucial steps in continuing the life cycle [1]. The timing of seed germination determines in which environment the seedling will grow and thus defines whether seedling establishment will be successful. Therefore, seeds need to sense the environment and initiate germination accordingly. Seed dormancy functions to time the moment of germination in the right (seasonal) environment. Seed dormancy is defined as the (temporary) inability of a viable seed to germinate under conditions otherwise favorable for germination [2]. Dormancy is thus not simply non-germination or quiescence. Dormancy also functions to spread germination in time, so that not all the offspring of one plant germinates at the same time and/or space [1,3]. There are different types of dormancy including physical dormancy, morphological dormancy, physiological dormancy, and combinational dormancy [1,4,5,6]. In certain types of physical dormancy, seeds have a hard, impermeable seed coat which has to be weakened first (e.g., by acids found in the digestive tract of an animal) before water can be taken up and germination can initiate. Morphological dormancy originates from an underdeveloped embryo, and the embryo requires time or a trigger to complete development, after which germination can occur. In the so-called physiological dormancy, the repression of seed germination upon seed imbibition, is maintained by living tissues, i.e., the embryo and the surrounding tissue, the endosperm. A combination of these different types of dormancy can also occur, e.g., morphophysiological dormancy. In this perspective, the focus will be on *Arabidopsis thaliana* which displays physiological dormancy [7]. A brief overview of secondary dormancy research in *A. thaliana* is provided, a working model is proposed, and a perspective on future research is given.

## 2. Primary Dormancy

Primary dormancy is the dormancy that is established during seed maturation on the mother plant. When mature, a dormant seed that is stored under dry conditions will lose its dormancy over time. This process is called seed after-ripening wherein largely unknown processes, likely linked to oxidation, allow the seed to gradually lose its dormancy (a phenomenon also referred to as “release” of seed dormancy), i.e., the seed gradually acquires the capacity to germinate (for a review, see [8]). During AR, reactive oxygen species (ROS) are non-enzymatically produced in sunflower seeds, releasing dormancy [9]. Upon imbibition, non-dormant *A. thaliana* seeds accumulate higher levels of ROS, indicating a signaling role for ROS during germination [10]. ROS thus play an important role in the regulation of both dormancy and germination [8]. The amount of dry storage time required to release dormancy can be used to define the dormancy levels stored in a seed. The level of primary dormancy is largely determined by the genetic background of the plant [11,12]. There is a large natural variation in the primary dormancy levels among *A. thaliana* accessions [13,14,15]. In addition, the maternal environment can modulate the level of seed dormancy in the progeny [16,17,18]. In particular, cold temperatures during seed development are well-known to increase seed dormancy levels (e.g., [18]).

Aside from dry storage, dormancy can also be released from the seed by several treatments on imbibed seeds. A common method is seed stratification, where imbibed seeds are exposed to low temperatures for a period of time prior to incubation at normal temperatures (e.g., [19]). Treatment with certain compounds, such as nitrate or karrikins (present in smoke), can also alleviate dormancy. These treatments have in common that they act on the hormone levels of gibberellins (promoting germination) or abscisic acid (ABA, repressing germination) or their signaling pathways [20]. Primary dormancy involves production of ABA by the endosperm and the embryo upon seed imbibition, while ABA catabolism is reduced and ABA sensitivity is increased compared to non-dormant seeds [7,20,21,22,23]. However, most of the current knowledge on dormancy comes from studies concerned with primary dormancy release in the dry seed [6,20,24,25].

## 3. Secondary Dormancy

When an imbibed non-dormant *A. thaliana* seed is exposed to certain prolonged unfavorable conditions, dormancy can be re-induced. This re-induced dormancy is termed secondary dormancy. Thus, importantly, secondary dormancy is not induced during seed maturation, and induction does not occur in dry seeds but rather in imbibed mature seeds [26]. Secondary dormancy induction occurs in nature when seeds cannot germinate due to the environmental conditions, e.g., when a seed becomes buried in the soil. In the field, dormancy cycling can be observed which is the seasonal induction and release of secondary dormancy [27,28]. The regulation of dormancy cycling in response to the natural environment has been extensively reviewed by Finch-Savage and Footitt [26]. Environmental signals that confer information about the seasonal changes are the temporal signals. Temperature is the main temporal signal controlling secondary dormancy cycling in the field [18,27,28,29]. It is known that the genetic background of the seeds controls secondary dormancy induction [3,30]. Genotypes that develop high primary dormancy levels during maturation also develop higher secondary dormancy levels upon re-induction of dormancy [28]. The repression of seed germination in secondary dormant seeds is poorly understood but most likely involves de novo ABA synthesis in *A. thaliana* [27]. However, other specific germination arrest mechanisms could be involved, as the mechanisms underlying secondary dormancy remain unclear. Nevertheless, an overlap between primary and secondary dormancy pathways likely takes place. Seeds that have not fully released their primary seed dormancy (see “assessing primary dormancy”) or accessions with higher natural primary seed dormancy levels might more efficiently enter a secondary dormant state [28,30,31].

## 4. Assessing Primary Dormancy

Seed dormancy is a functionally defined trait, i.e., it requires a germination test to determine whether a seed is dormant or not. However, germination may or may not be observed depending on which favorable germination conditions are used for the test. This is because seeds lose dormancy gradually, i.e., they gradually acquire the capacity to germinate under suboptimal, but still favorable, germination conditions. Hence, after a certain amount of after-ripening time, a given seed batch may not germinate under favorable germination conditions A but may fully germinate under favorable germination conditions B where conditions A are less favorable than conditions B (e.g., [32]). It follows that the same seed batch can be non-dormant or dormant depending on the particular favorable germination assay being used [20]. In this case, one may say that the seed batch contains residual primary dormancy or that it has not fully released its dormancy.

## 5. Assessing Secondary Dormancy

In the laboratory, there are a number of methods available for the induction of secondary dormancy in *A. thaliana* [29,30,31,33,34,35]. Typically, imbibed seeds are stored in the dark, and temperature and/or osmotic stress treatments are used to induce secondary dormancy. The Col-0 genotype is often used in *A. thaliana* research, for which mutant lines are conveniently available through stock centers. However, Col-0 is an accession which naturally has low primary seed dormancy levels. Therefore, it is likely that in Col-0, secondary dormancy induction is difficult to observe, as in accessions with low primary seed dormancy levels, secondary dormancy induction is slower [28,30]. Using seeds with residual primary dormancy levels, as defined above, aids secondary dormancy induction (e.g., [30]). As is the case for primary dormancy, secondary dormancy may be revealed by adjusting the conditions of the germination assay. Hence, a seed batch that underwent a secondary dormancy inducing treatment may fully germinate under favorable germination conditions B but may not germinate under favorable germination conditions A, where conditions A are less favorable than conditions B. Hence, germination conditions A are more suited to detect secondary dormancy. Increasing the temperature used in the germination assay has proven to be a useful approach to reveal shallow primary and secondary dormancy in *A. thaliana*, barley, and tomato seeds [36,37,38]. Secondary dormancy is more easily induced in accessions that naturally have higher primary dormancy levels, such as Landsberg *erecta* (L*er*) and, especially, Cape Verde Islands (Cvi). Therefore, these accessions might be more suitable to study (secondary) dormancy [13].

Secondary dormancy can also be studied in field experiments with *A. thaliana*, which most certainly mimic natural dormancy cycling [3,27,28]. When after-ripened, i.e., non-dormant, seeds are buried in the field, secondary dormancy is induced during autumn and winter, and released in spring [27,28]. Secondary dormancy induction in the field requires months and may involve a progressive decrease in temperatures [27,28]. It might be that the mechanisms responsible for secondary dormancy induction intrinsically requires extended periods of time in conjunction with gradual decrease in temperature. Thus, these mechanisms might be difficult to recreate under laboratory conditions under shorter periods of time. Therefore, field experiments may become indispensable for secondary dormancy research, albeit not the most practical. Due to regulations, a field study with mutants might not be possible in some countries, and there might be other practical difficulties. Field experiments are also possible with larger populations [3,18,27,28].

When conducting secondary-dormancy-related experiments, including multiple time-points to assess dormancy levels during release or induction allows to monitor how dormancy develops during the experiment. An example of this is the simulated dormancy cycling described by Footitt et al. [34]. It remains to be understood whether laboratory protocols induce secondary dormancy with the same underlying mechanisms as secondary dormancy induction by field conditions. Indeed, most laboratory studies use protocols that involve transferring imbibed seeds from cold temperatures to hot temperatures, which triggers the secondary dormancy induction. This is, however, the opposite in the field, where dormancy is induced during the cold periods in winter and released by the increase in the seasonal temperature. It is expected that the field experiments reflect natural conditions, and therefore, mimicking those conditions in the laboratory is of paramount importance for the study of seed secondary dormancy.

## 6. Different Ecological Functions of Primary and Secondary Dormancy

Dormancy functions to time the moment of germination in accordance with the environment to increase the chance of successful subsequent seedling establishment. Primary and secondary dormancy both share this function; however, they interact with different environments. The maternal environment is a large factor in determining the primary dormancy level. Thus, primary dormancy confers information about the environment as experienced by the mother plant. A suitable maternal environment, i.e., optimal maternal growth conditions (in terms of temperature, light, and nutrient availability) result in seeds with lower primary dormancy [16]. This might favor seed germination directly after shedding and can result in another growth cycle in this suitable environment [39]. Over time, seeds may lose their primary dormancy but may not germinate due to the absence of imbibition or imbibition in the presence of unfavorable germination conditions. In this case, non-germinated seeds form the seed soil bank, which is the realm where processes regulating secondary dormancy become predominant. Under these conditions, it is expected that the immediate environmental conditions, rather than those experienced by the mother plant, dominate to determine secondary dormancy levels. Indeed, previous reports seem to support this notion [3,28]. However, this is in contrast with previous reports where the effect of the maternal environment on primary dormancy influences also the secondary dormancy status [30,31,35]. It might be the case that residual primary dormancy in the seeds in these studies explains the observed differences. Both in laboratory studies and in nature, primary dormancy might not be fully released prior to secondary dormancy induction. Therefore, it is important to separate the two, and as long as it is unknown how similar both are, this has to be better defined.

## 7. Overlap and Differences between Primary and Secondary Dormancy

Although the level of overlap is unclear, it can be safely assumed that primary and secondary dormancy pathways converge to activate the ABA-dependent processes that repress seed germination in imbibed seeds. Consistent with this notion, an early laboratory study comparing the transcriptome changes during primary dormancy, dormancy release, and secondary dormancy induction found that primary and secondary dormant states had an over-representation of ABA-responsive genes [33]. Primary and secondary dormancy are more likely to differ in how the seeds sense environmental conditions to regulate dormancy. Indeed, the environment experienced by the developing seed or the after-ripening dry seed, which influences primary seed dormancy levels, is not the same as that experienced by the seed in the soil bank. The case of the DELAY OF GERMINATION 1 (DOG1) protein in *A. thaliana* might indeed support this view. DOG1 is a protein of unknown function that promotes dormancy, since mutating DOG1 strongly reduces primary dormancy levels [40]. DOG1 levels in primary dormant seeds correlate with dormancy levels, but DOG1 levels do not decrease during after-ripening. It is likely that that DOG1 is oxidized during after-ripening and loses its activity to inhibit germination [41]. This indicates that it is a target of the pathways sensing the environment during dry after-ripening. However, the *dog1-2* mutant displays a reduced but not absent secondary dormancy induction [34]. Surprisingly, a recent study reported that the *DOG1* gene is not located in any secondary dormancy associated Quantitative Trait Locus (QTL), although there is a QTL next to the *DOG1* locus, *SET1* (*Seedling Emergence Timing 1*, which reflects dormancy cycling) [18]. These data therefore indicate that seeds may utilize different sensory pathways to regulate primary and secondary dormancy but do not exclude that they also utilize the same pathways.

## 8. A Model for the Regulation of Secondary Dormancy

**Figure 1:**
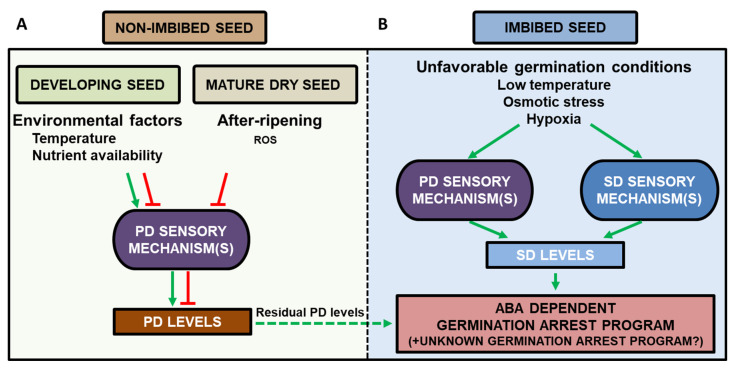
A working model connecting primary (PD) and secondary (SD) dormancy. (**A**) Seed development and the dry seed. The maternal environmental factors are perceived by the PD sensory mechanism(s), which determines final PD levels stored in the mature dry seed. In the mature dry seed, after-ripening processes take place, likely involving accumulation of oxidative events, lowering the PD levels. (**B**) Establishment of SD in the imbibed seed can take place in non-dormant seeds, i.e., seeds that germinate under particular, favorable seed germination conditions. Upon imbibition, continuous exposure to unfavorable environmental factors is perceived in an unknown manner. This could involve dedicated SD sensory mechanism(s) and/or the PD sensory mechanisms operating in the developing seed. In turn, the sensory mechanism promotes an increase in SD levels that activate germination arrest program(s), so that germination does not take place even under favorable germination conditions. Residual PD levels may promote the onset of SD upon seed imbibition (dashed line). Seeds may cycle between non-dormant and SD.

Ultimately, the germination arrest response observed in both primary and secondary dormancy is the result of internal signaling pathways responding to environmental factors. Primary dormancy levels are established during seed maturation and are determined by the genetic background of the seed and the maternal environmental parameters such as the temperature during seed development (Figure 1A). Upon imbibition of a dormant seed, the primary seed dormancy levels present in the seed activate the germination arrest program controlled by ABA (Figure 1B). The after-ripening processes in dry seeds that reduce primary dormancy levels are thought to mainly involve oxidative processes [8,20]. Eventually, oxidative events occurring during the after-ripening period reduce primary seed dormancy levels so that the germination arrest program is no longer activated under a given favorable seed germination condition (Figure 1).

Secondary dormancy can be induced by imbibing seeds under unfavorable environmental conditions for a prolonged period of time, such as low temperatures or osmotic stress (Figure 1B). Environmental factors inducing secondary dormancy levels may act in two manners that do not exclude each other: (1) they may be perceived by the same sensory mechanisms that regulate primary seed dormancy levels during seed maturation or (2) they may be perceived by dedicated secondary dormancy sensory mechanisms (Figure 1B). The nature of the sensory mechanisms remains to be determined. Recent reviews discuss possible mechanisms and players in sensing the environment. DOG1 is hypothesized to play a role in the sensing of temperature in the imbibed seed, the main factor driving dormancy cycling [18,26,28,34]. Oxygen and ROS signaling might confer information about the environment [8,26]. Hypoxia occurs when the soil becomes waterlogged for example, and ROS are signaling molecules for a number of stresses. Light, or the absence of light, interacts with hormonal levels [26,42]. Nutrient availability and osmotic conditions confer information about the suitability of the germination environment [26]. Finally, the organelles function in ABA production and might play a role in dormancy signaling [28,43].

The sensory mechanism integrates the environmental signals and induces secondary dormancy levels that stimulate the germination arrest program controlled by ABA and possibly an additional germination arrest program specifically acting during secondary dormancy establishment. As for primary dormancy levels, the molecular nature of secondary dormancy levels remains unknown. Primary dormancy levels might still be present and potentially detectable under less favorable germination conditions. In the proposed model, residual primary seed dormancy levels may further activate the seed germination arrest programs and thus facilitate the onset of secondary seed dormancy upon seed imbibition (Figure 1A,B dashed line). It remains unknown how primary and secondary dormancy levels control the germination arrest program (Figure 1B) [20]. However, certain central proteins and processes in the regulation of dormancy have been identified or hypothesized. A central transcription factor in ABA signaling is ABA-Insensitive 5 (ABI5), integrating stress signaling and other phytohormones [23,44]. DOG1, a protein of yet unknown function, is a major player in the regulation of primary dormancy [23,45].

## 9. Perspectives

Secondary dormancy studies in *A. thaliana* will shed a light on the molecular mechanisms. It is unclear to which extent these mechanisms overlap with those of primary dormancy. A clear distinction between primary and secondary dormancy in experiments is therefore essential. These experiments would benefit from a protocol that is confirmed to mimic natural dormancy cycling. One way to identify the overlap between artificial methods and a field burial experiment is to compare RNAseq data of the same genotype in both a laboratory setting as field setting. For example, the protocol described by Footitt et al., 2017 [34], could be used to mimic dormancy cycling of Cvi or L*er* seeds, of which the RNAseq data could then be compared to the RNAseq data that recently became available for these genotypes [18,28]. Such a protocol could be used to study some of the remaining questions. (1) The level and nature of the epigenetic control of dormancy cycling—It is known that there is epigenetic control of primary dormancy, and the mechanisms are being revealed [25,46,47]. There is also likely epigenetic control of dormancy cycling [29]; therefore, studying (genome-wide) epigenetics during dormancy cycling is an interesting next step. (2) What is the role of translation and translational control during dormancy cycling? Considering the large changes occurring in the expression of the genes associated with the translational machinery during dormancy cycling [28], and as most of the knowledge on dormancy originates from studies on dry seeds, this is a vast unknown. Finally, the dynamic and adaptive nature of dormancy cycling might provide novel insights in the mechanisms for both primary and secondary dormancy.

## References

[1] Bewley J.D., Bradford K.J., Hilhorst H.W.M., Nonogaki H. (2013). Seeds: Physiology of Development, Germination and Dormancy.

[2] Benech-Arnold R.L., Sánchez R.A., Forcella F., Kruk B.C., Ghersa C.M. (2000). Environmental control of dormancy in weed seed banks in soil. Field Crop. Res..

[3] Postma F.M., Lundemo S., Ågren J. (2016). Seed dormancy cycling and mortality differ between two locally adapted populations of Arabidopsis thaliana. Ann. Bot..

[4] Nikolaeva M.G., Khan A.A. (1977). Factors controlling the seed dormancy pattern. The Physiology and Biochemistry of Seed Dormancy.

[5] Baskin J.M., Baskin C.C. (2004). A classification system for seed dormancy. Seed Sci. Res..

[6] Finch-Savage W.E., Leubner-Metzger G. (2006). Seed dormancy and the control of germination. New Phytol..

[7] Lee K.P., Piskurewicz U., Turečková V., Strnad M., Lopez-Molina L. (2010). A seed coat bedding assay shows that RGL2-dependent release of abscisic acid by the endosperm controls embryo growth in Arabidopsis dormant seeds. Proc. Natl. Acad. Sci. USA.

[8] Bailly C. (2019). The signalling role of ROS in the regulation of seed germination and dormancy. Biochem. J..

[9] Oracz K., El-Maarouf Bouteau H., Farrant J.M., Cooper K., Belghazi M., Job C., Job D., Corbineau F., Bailly C. (2007). ROS production and protein oxidation as a novel mechanism for seed dormancy alleviation. Plant J..

[10] Leymarie J., Vitkauskaité G., Hoang H.H., Gendreau E., Chazoule V., Meimoun P., Corbineau F., El-Maarouf-Bouteau H., Bailly C. (2012). Role of reactive oxygen species in the regulation of arabidopsis seed dormancy. Plant Cell Physiol..

[11] Alonso-Blanco C., Hanhart C.J., Blankestijn-de Vries H., Koornneef M. (2003). Analysis of natural allelic variation at seed dormancy loci of Arabidopsis thaliana. Genetics.

[12] Clerkx E.J.M., El-Lithy M.E., Vierling E., Ruys G.J., Blankestijn-De Vries H., Groot S.P.C., Vreugdenhil D., Koornneef M. (2004). Analysis of natural allelic variation of Arabidopsis seed germination and seed longevity traits between the accessions Landsberg erecta and Shakdara, using a new recombinant inbred line population. Plant Physiol..

[13] Bentsink L., Soppe W., Koornneef M. (2007). Genetic aspects of seed dormancy. Annu. Plant Rev..

[14] Atwell S., Huang Y.S., Vilhjálmsson B.J., Willems G., Horton M., Li Y., Meng D., Platt A., Tarone A.M., Hu T.T. (2010). Genome-wide association study of 107 phenotypes in Arabidopsis thaliana inbred lines. Nature.

[15] Vidigal D.S., Marques A.C.S.S., Willems L.A.J., Buijs G., Méndez-Vigo B., Hilhorst H.W.M., Bentsink L., Picó F.X., Alonso-Blanco C. (2016). Altitudinal and climatic associations of seed dormancy and flowering traits evidence adaptation of annual life cycle timing in Arabidopsis thaliana. Plant Cell Environ..

[16] He H., De Souza Vidigal D., Basten Snoek L., Schnabel S., Nijveen H., Hilhorst H., Bentsink L. (2014). Interaction between parental environment and genotype affects plant and seed performance in Arabidopsis. J. Exp. Bot..

[17] Springthorpe V., Penfield S. (2015). Flowering time and seed dormancy control use external coincidence to generate life history strategy. eLife.

[18] Footitt S., Walley P.G., Lynn J.R., Hambidge A.J., Penfield S., Finch-Savage W.E. (2019). Trait analysis reveals DOG1 determines initial depth of seed dormancy, but not changes during dormancy cycling that result in seedling emergence timing. New Phytol..

[19] Buijs G., Kodde J., Groot S.P.C., Bentsink L. (2018). Seed dormancy release accelerated by elevated partial pressure of oxygen is associated with DOG loci. J. Exp. Bot..

[20] Chahtane H., Kim W., Lopez-Molina L. (2017). Primary seed dormancy: A temporally multilayered riddle waiting to be unlocked. J. Exp. Bot..

[21] Ali-Rachedi S., Bouinot D., Wagner M.H., Bonnet M., Sotta B., Grappin P., Jullien M. (2004). Changes in endogenous abscisic acid levels during dormancy release and maintenance of mature seeds: Studies with the Cape Verde Islands ecotype, the dormant model of Arabidopsis thaliana. Planta.

[22] Millar A.A., Jacobsen J.V., Ross J.J., Helliwell C.A., Poole A.T., Scofield G., Reid J.B., Gubler F. (2006). Seed dormancy and ABA metabolism in Arabidopsis and barley: The role of ABA 8’-hydroxylase. Plant J..

[23] Nonogaki H. (2019). ABA responses during seed development and germination. Adv. Bot. Res..

[24] Née G., Xiang Y., Soppe W.J. (2017). The release of dormancy, a wake-up call for seeds to germinate. Curr. Opin. Plant Biol..

[25] Nonogaki H. (2017). Seed biology updates—Highlights and new discoveries in seed dormancy and germination research. Front. Plant Sci..

[26] Finch-Savage W.E., Footitt S. (2017). Seed dormancy cycling and the regulation of dormancy mechanisms to time germination in variable field environments. J. Exp. Bot..

[27] Footitt S., Douterelo-Soler I., Clay H., Finch-Savage W.E. (2011). Dormancy cycling in Arabidopsis seeds is controlled by seasonally distinct hormone-signaling pathways. PNAS.

[28] Buijs G., Vogelzang A., Nijveen H., Bentsink L. (2019). Dormancy cycling: Translation related transcripts are the main difference between dormant and non-dormant seeds in the field. Plant J..

[29] Footitt S., Müller K., Kermode A.R., Finch-Savage W.E. (2015). Seed dormancy cycling in Arabidopsis: Chromatin remodelling and regulation of DOG1 in response to seasonal environmental signals. Plant J..

[30] Penfield S., Springthorpe V. (2012). Understanding chilling responses in Arabidopsis seeds and their contribution to life history. Philos. Trans. R. Soc. B Biol. Sci..

[31] Auge G.A., Blair L.K., Burghardt L.T., Coughlan J., Edwards B., Leverett L., Donohue K. (2015). Secondary dormancy dynamics depends on primary dormancy status in Arabidopsis thaliana. Seed Sci. Res..

[32] De Giorgi J., Piskurewicz U., Loubery S., Utz-Pugin A., Bailly C., Mène-Saffrané L., Lopez-Molina L. (2015). An endosperm-associated cuticle is required for Arabidopsis seed viability, dormancy and early control of germination. PLoS Genet..

[33] Cadman C.S.C., Toorop P.E., Hilhorst H.W.M., Finch-Savage W.E. (2006). Gene expression profiles of Arabidopsis Cvi seeds during dormancy cycling indicate a common underlying dormancy control mechanism. Plant J..

[34] Footitt S., Ölçer-Footitt H., Hambidge A.J., Finch-Savage W.E. (2017). A laboratory simulation of Arabidopsis seed dormancy cycling provides new insight into its regulation by clock genes and the dormancy-related genes DOG1, MFT, CIPK23 and PHYA. Plant Cell Environ..

[35] Coughlan J.M., Saha A., Donohue K. (2017). Effects of pre- and post-dispersal temperature on primary and secondary dormancy dynamics in contrasting genotypes of Arabidopsis thaliana (Brassicaceae). Plant Species Biol..

[36] Geshnizjani N., Ghaderi-Far F., Willems L.A.J., Hilhorst H.W.M., Ligterink W. (2018). Characterization of and genetic variation for tomato seed thermo-inhibition and thermo-dormancy. BMC Plant Biol..

[37] Nagel M., Alqudah A.M., Bailly M., Rajjou L., Pistrick S., Matzig G., Börner A., Kranner I. (2019). Novel loci and a role for nitric oxide for seed dormancy and preharvest sprouting in barley. Plant Cell Environ..

[38] Footitt S., Clewes R., Feeney M., Finch-Savage W.E., Frigerio L. (2019). Aquaporins influence seed dormancy and germination in response to stress. Plant Cell Environ..

[39] Burghardt L.T., Metcalf C.J.E., Wilczek A.M., Schmitt J., Donohue K. (2015). Modeling the influence of genetic and environmental variation on the expression of plant life cycles across landscapes. Am. Nat..

[40] Bentsink L., Jowett J., Hanhart C.J., Koornneef M. (2006). Cloning of DOG1, a quantitative trait locus controlling seed dormancy in Arabidopsis. Proc. Natl. Acad. Sci. USA.

[41] Nakabayashi K., Bartsch M., Xiang Y., Miatton E., Pellengahr S., Yano R., Seo M., Soppe W.J.J. (2012). The time required for dormancy release in Arabidopsis is determined by DELAY OF GERMINATION1 protein levels in freshly harvested seeds. Plant Cell.

[42] Seo M., Nambara E., Choi G., Yamaguchi S. (2009). Interaction of light and hormone signals in germinating seeds. Plant Mol. Biol..

[43] Nonogaki H. (2019). The long-standing paradox of seed dormancy unfolded?. Trends Plant Sci..

[44] Skubacz A., Daszkowska-Golec A., Szarejko I. (2016). The role and regulation of ABI5 (ABA-insensitive 5) in plant development, abiotic stress responses and phytohormone crosstalk. Front. Plant Sci..

[45] Soppe W.J.J., Bentsink L. (2020). Seed dormancy back on track; its definition and regulation by DOG1. New Phytol..

[46] Nonogaki H. (2014). Seed dormancy and germination-emerging mechanisms and new hypotheses. Front. Plant Sci..

[47] Iwasaki M., Hyvarinen L., Piskurewicz U., Lopez-Molina L. (2019). Non-canonical RNA-directed DNA methylation participates in maternal and environmental control of seed dormancy. eLife.

